# Characteristics and outcome of the COEUR Canadian validation cohort for ovarian cancer biomarkers

**DOI:** 10.1186/s12885-018-4242-8

**Published:** 2018-03-27

**Authors:** Cécile Le Page, Kurosh Rahimi, Martin Köbel, Patricia N. Tonin, Liliane Meunier, Lise Portelance, Monique Bernard, Brad H. Nelson, Marcus Q. Bernardini, John M. S. Bartlett, Dimcho Bachvarov, Walter H. Gotlieb, Blake Gilks, Jessica N. McAlpine, Mark W. Nachtigal, Alain Piché, Peter H. Watson, Barbara Vanderhyden, David G. Huntsman, Diane M. Provencher, Anne-Marie Mes-Masson

**Affiliations:** 1 Centre de recherche du Centre hospitalier de l’Université de Montréal (CRCHUM) and Institut du cancer de Montréal, Montreal, QC Canada; 2 Department of Pathology du Centre hospitalier de l’Université de Montréal, Montreal, QC Canada; 3 Department of Pathology and Laboratory of Medicine, University of Calgary, Calgary, AB Canada; 4 Departments of Medicine and Human Genetics, McGill University; Cancer Research Program, The Research Institute of the McGill University Health Centre, Montreal, QC Canada; 5 Tumour Tissue Repository, Trev and Joyce Deeley Research Centre, BC Cancer Agency, Victoria, BC Canada; 6 Department of Obstetrics and Gynaecology, University of Toronto, Toronto, ON Canada; 7 Diagnostic Development and Ontario Tumour Bank, Ontario Institute for Cancer Research, Toronto, ON Canada; 8 Centre de recherche du CHU de Quebec, Quebec, QC Canada; 9 Laboratory of Gynecologic Oncology, Lady Davis Research Institute, Jewish General Hospital, Montreal, QC Canada; 10 Department of Pathology, Vancouver General Hospital and University of British Columbia, Vancouver, BC Canada; 11 Department of Obstetrics and Gynaecology, University of British Columbia, Vancouver, BC Canada; 12 Department of Biochemistry & Medical Genetics, University of Manitoba, Winnipeg, MB Canada; 13 Centre de Recherche du CHUS. Département de Microbiologie et Infectiologie, Faculté de Médecine, Université de Sherbrooke, Sherbrooke, Canada; 14 Cancer Therapeutics Program, Ottawa Hospital Research Institute, Ottawa, Canada; 15 Department of Cellular and Molecular Medicine, University of Ottawa, Ottawa, Canada; 16 Division of Gynecologic Oncology, Université de Montréal, Montreal, Canada; 17 Department of Medicine, Université de Montréal, Montreal, Canada; 18 900 rue Saint Denis, Tour R, Montreal, H2X2A0 Canada

**Keywords:** Epithelial ovarian cancer, Histotype, Biomarker, BRCA, Survival, Treatment response

## Abstract

**Background:**

Ovarian carcinoma is the most lethal gynecological malignancy due to early dissemination and acquired resistance to platinum-based chemotherapy. Reliable markers that are independent and complementary to clinical parameters are needed to improve the management of patients with this disease. The Canadian Ovarian Experimental Unified Resource (COEUR) provides researchers with biological material and associated clinical data to conduct biomarker validation studies. Using standards defined by the Canadian Tissue Repository Network (CTRNet), we have previously demonstrated the quality of the biological material from this resource. Here we describe the clinical characteristics of the COEUR cohort.

**Methods:**

With support from 12 Canadian ovarian cancer biobanks in Canada, we created a central retrospective cohort comprised of more than 2000 patient tissue samples with associated clinical data, including 1246 high-grade serous, 102 low-grade serous, 295 endometrioid, 259 clear cell and 89 mucinous carcinoma histotypes. A two-step reclassification process was applied to assure contemporary histological classification (histotyping). For each histotypes individually, we evaluated the association between the known clinico-pathological parameters (stage, cytoreduction, chemotherapy treatment, *BRCA1* and *BRCA2* mutation) and patient outcome by using Kaplan-Meier and Cox proportional hazard regression analyses.

**Results:**

The median follow-up time of the cohort was 45 months and the 5-year survival rate for patients with high-grade serous carcinomas was 34%, in contrast to endometrioid carcinomas with 80% at 5 years. Survival profiles differed by histotype when stratified by stage or cytoreduction. Women with mucinous or clear cell carcinomas at advanced stage or with non-optimally debulked disease had the worst outcomes. In high-grade serous carcinoma, we observed significant association with longer survival in women harboring *BRCA1 or BRCA2* mutation as compared to patients without detectable mutation.

**Conclusions:**

Our results show the expected survival rates, as compared with current literature, in each histotype suggesting that the cohort is an unbiased representation of the five major histotypes. COEUR, a one stop comprehensive biorepository, has collected mature outcome data and relevant clinical data in a comprehensive manner allowing stratified analysis.

## Background

Although epithelial ovarian cancer (EOC) has an incidence rate ten times less frequent than breast cancer, is the fifth-leading cause of cancer-related deaths among women in the Western world [1]. EOC encompasses at least five distinct diseases, represented by five histopathological types, herein designate as histotypes, with unique characteristics, different molecular features, different clinical behavior, and sites of origin: high-grade serous carcinoma (HGSC), low-grade serous carcinoma (LGSC), endometrioid carcinoma (EC), mucinous carcinoma (MC), and clear cell carcinoma (CCC) [2, 3]. The less common histotypes LGSC and MC, could be considered rare diseases with approximately 1000 cases per year in the US [1]. HGSC is the most common histotype and is often diagnosed at an advanced stage (stage III or IV) purportedly due its rapid growth rate, origin in the fallopian tube, its associated with local spread into the peritoneal cavity, and late onset of symptoms. This is in contrast to other histotypes, which often present as a localized pelvic mass. The standard treatment for EOC, particularly for HGSC, is debulking surgery and platinum-based chemotherapy. However, resistance to chemotherapy often develops, contributing to a low 5-year survival rate for patients with advanced stage disease [1]. Assessment of clinical parameters, such as histotype, disease stage and residual disease (RD), are important factors in determining the management of patients, but they cannot always predict response to treatment and survival. Although the length of survival has significantly improved in the last decade with recent discoveries of new therapeutic targets and drug developments, survival rates and the quality of life of patients remains poor [4]. Therefore, reliable markers that are independent and complementary to clinical parameters are needed to improve patient management.

Diseases as heterogeneous as EOC require the support of large research networks to provide access to enough specimens to investigate the association between linked clinical data and deep molecular analysis for the development of precision medicine. For this reason, the Australian Ovarian Cancer Study was launched in 2001 and has accrued more than 2000 cases in an attempt to identify genetic variants that can be associated with the development of ovarian cancer (http://www.aocstudy.org). In UK, BriToc is collecting tumor samples at diagnosis and recurrence (http://ovarian.org.uk/our-research/the-research-we-fund/) in four academic centers. Likewise, the Canadian Ovarian Experimental Unified Resource (COEUR) program, the first Canadian project to be initiated by the Ovarian Cancer Consortium, formed in 2009, with the collaboration of the GOC (Society of Gynecologic Oncology of Canada), and a group of investigators and biobank scientists established the program to develop a high-quality biospecimen repository with associated clinical data for large-scale collaborations that could advance research in molecular biomarker validation in EOC (http://www.tfri.ca/en/research/translational-research/coeur.aspx). Importantly, this resource is available to researchers through a managed review process for approved ovarian cancer studies at: http://www.tfri.ca/en/research/translational-research/coeur/coeur_access.aspx. The main goal of the COEUR focuses on biomarker validation for more accurate classification of pathological specimens and better prediction of the clinical outcomes. Taking advantage of the pre-existing ovarian cancer biobanks in Canada, we created a central retrospective cohort of patient tissue samples. The feasibility of this pan-Canadian ovarian cancer repository and biomarker program was assessed in the pilot phase of the project that was completed in 2010. The main activities were centralized at the Centre de recherche du Centre hospitalier de l’Université de Montréal (CRCHUM, Montreal, QC), and involved the small collection of specimens with associated data, and included processing and quality control of samples for use in research assays [5]. We demonstrated that an adequate resource was available to create a national cohort for the validation of molecular biomarkers in ovarian cancer. Moreover, this pilot study allowed the COEUR to proceed with confidence in collecting 2000 retrospective cases and enter the next phase of the program.

In the second phase of the COEUR program we collected DNA from normal fallopian tube or buffy coat, FFPE tissues, frozen tissues, ascites, plasma and serum when available. The initial goal was to collect samples from the five main histotypes: HGSC, EC, MC, LGSC and CCC. HGSC represented the majority of collected cases, whereas MC and LGSC cases were limited to at least 100 cases. Because before 2015, local ovarian cancer diagnosis showed up to 15% of misclassification, here two pathologists, one at CRCHUM and one at the University of Calgary, performed a double central review of FFPE blocks.

The present study aimed to describe the characteristics and clinico-pathological prognostic factors of the pan-Canadian COEUR cohort. Because different histopathologies of ovarian cancer are now recognized as distinct diseases, we analyzed these parameters separately for each histotype. Combined with associated clinical data, we show that the survival rates reflect the different clinical behavior of these diseases [2].

## Methods

### Participating biobanks

The participating biobanks of the COEUR program are described in Table 1. A material transfer agreement was signed in 2010 by all participating biobanks, the Terry Fox Research Institute and the CRCHUM, to centralize and distribute samples and their associated clinical data for approved research. Between 2010 and 2017, a total of 2045 retrospective cases were collected and shipped to the central location, the CRCHUM.

**Table 1:** Description of biobanks participating in the COEUR program

Bank Name*	Location (Canada)*	Established in	Funding or support*	Partner*	Web address
Segal Cancer Center Lady Davis Institute biobank	Lady Davis Institute-Jewish General Hospital, Montreal, QC	2003	Fonds de la recherche en santé Québec (network RRCancer), Montreal-Israel Cancer Research Foundation, thee Gloria’s Girls Fund, the Susan and Jonathn Wener fund and the Garber fund.	CTRNet, Ovarian Cancer Canada	www.rrcancer.ca
Banque de tissus et de données du Réseau de recherche sur le cancer du FRQS	CRCHUM-Montreal, QC	2000	Fonds de la recherche en santé Québec (network RRCancer), Ovarian Cancer Canada	CTRNet	www.rrcancer.ca
Ottawa Ovarian Cancer Tissue Bank	Ottawa Hospital Research Institute, Ottawa, ON	1995	OCC, Ottawa Hospital	Ovarian Cancer Canada	N/A
OVCARE Gynecologic Tissue Bank	Vancouver General Hospital, Vancouver, BC	2001	OVCARE BC, Michael Smith Foundation for Health Research and Ovarian Cancer Canada	BC Cancer Agency, Vancouver Coastal Health Research Institute, University BC, Vancouvver General Hospital	www.ovcare.ca/research/platforms.php
Ovarian cancer specimen and data bank at CHUQ	Hopital Hotel-Dieu de Quebec, Quebec, QC	2000	Fonds National de Recherche du Québec (network RRCancer)	Fonds de la recherche en santé Québec (network RRCancer), CTRNet	www.rrcancer.ca
Banque d’échantillons biologiques et de données de Sherbrooke	Faculté de recherche et des sciences de la santé, Université de Sherbrooke, Sherbrooke, QC	1998	Fonds de la recherche en santé Québec (RRCancer) and Quebec breast cancer foundation	CTRNet, Ovarian Cancer Canada	www.rrcancer.ca
Alberta Cancer Research Biobank	University of Alberta, Edmonton, AB; and Univesity of Calgary, Calgary, AB	2001	Alberta Cancer Foundation, Canadian Breast Cancer foundation	CTRNet, Canadian Breast Cancer foundation	http://www.acrb.ca/
BC Cancer Agency Tumour Tissue Repository	BC Cancer, Agency, Vancouver Island Center, BC	2004	BC Cancer Agency and Provinical Health Services Authority	CTRNet	www.bccrc.ca/dept/ttr
UHN BioBank	Toronto General Hospital	2013	UHN Research Institute, UHN Foundation	N/A	https://www.uhnresearch.ca/service/uhn-biospecimen-sciences-program-bsp
UHN program in Biospecimen Sciences	Sunnybrook Hospital, Toronto, ON	2001	UHN Research Institute, UHN Foundation	N/A	N/A
Ontario Tumour Bank	Ontario Institute for Cancer Research MaRS Centre, Toronto, ON	2004	Ontario Institute for Cancer Research	N/A	ontariotumourbank.ca
Manitoba Ovarian Biobanking Program	University of Manitoba, Winnipeg, MN	2010	N/A	CTRNet	N/A

*Abbreviations: *CHUQ* Centre Hospitalier Universitaire de Quebec, *CRCHUM* Centre de Recherche du CHUM, *CRTNet* Canadian Tissue Repository Network; recherche du Quebec, *UHN* University Health Network

### Selection criteria and specimen collection

The initial selection criteria included patients with no chemotherapy treatment before oophorectomy (neoadjuvent treatment) to avoid potential effects on protein expression by the chemotherapeutic treatment and subsequent bias towards biomarker validation. However, 100 cases with neoadjuvant treatment were collected before full clinical data was assessed and passed through this filter and were included in the cohort. Inclusion criteria also required a minimum 12-month follow-up if there was no event of recurrence or death. This criteria was not verified by all biobanks: we received 55 samples with little (< 6 months) or no follow-up time after diagnosis. For biospecimens, selection criteria included either frozen or FFPE tissues, and if available, DNA from non-cancerous tissues, serum, ascites, or plasma. The last criteria involved the exclusion of the following histotypes: borderline tumors, mixed histotypes, Brenner or undifferentiated carcinomas. Specimens (*n* = 2045) that were received at the CHUM central location were re-evaluated under the selection criteria described above. In addition, the COSPv3 8-immunohistochemical marker panel in conjunction with re-review arbitration (when necessary) was applied to confirm or reclassify the histotype diagnosis of each case. Twenty-nine cases (2.4%) could not be assigned to one of the five histotypes (i.e Brenner tumor, not primary ovarian or undifferentiated). Twenty-five cases did not satisfy the selection criteria because only borderline tumor tissue was found on the sample received. Six cases could not be assigned to a histotype because FFPE blocks were not available for additional analysis, and the original diagnosis did not clearly indicate whether they were LGSC or HGSC cases.

### Clinical data collection and definition of parameters

In accordance with the ethics approval and material transfer agreement, only anonymized data was transferred from the source biobank to the central location at the CRCHUM (Montreal, QC, Canada). A template spreadsheet standardized the collection of data and minimized variation in data submission across participating institutions. Data were reviewed by a scientist in consultation with the clinical data monitoring committee, comprised of two gynecologic oncologists who identified potentially inaccurate or non-standardized data. Spreadsheets were reviewed for discrepancies or missing data, and participating biobanks were contacted to correct inconsistant data.

Clinical data were updated annually for follow-up. In December 2017, the 5-year survival rate for the entire cohort was 45%, which corresponds with the overall survival rate reported for ovarian carcinoma [1]. Cases with no follow-up were not included in the overall survival analysis. Long-term survivors were defined as patients who survived at least 10 years after diagnosis. Patients with less than 10 years follow-up and no record of death specific to ovarian cancer were not included in the long-term survivor proportion indicated in Table 2. The amount of residual disease (RD) or cytoreduction efficiency was difficult to collect due to lack of consistent reporting in the biobank data (66%). To standardize this data we translated the RD amount as non-optimal cytoreduction when RD was > 1 cm as per 2012 clinical standards [6]. When only ‘optimal’ cytoreduction was collected from biobanks, we could not translate this information into the measurable variable (> 1 cm or 1–2 cm) since the standard has changed over time and also may not be the same in all institutions.

**Table 2:** Patient Characteristics of the COEUR cohort

Histotypes		HGSC	EC	LGSC	CCC	MC	Total
	Number of samples per histotypes	1246	296	102	259	88	1991
Age diagnosis (years)	Mean	62	58	53	56	53	60
Age end of follow-up (years)	Mean	66	63	59	61	58	64
Follow-up time (months)	Mean	47	64	60	58	52	51
	Min-Max	0–211	0–268	0–270	0–247	1–228	0–270
	Total *N*	1240	294	102	258	87	1983
Stage	1	70(6%)	145(54%)	13(14%)	127(52%)	61(74%)	416
	2	137(12%)	74(28%)	7(7%)	54(22%)	11(13%)	283
	3	835(72%)	45(17%)	67(72%)	58(23%)	8(9%)	1013
	4	123(11%)	5(2%)	6(6%)	7(3%)	3(3%)	144
	Total *N*	1165	269	93	246	83	1856
Residual Disease	none	207(28%)	120(75%)	16(22%)	93(68%)	36(81%) 472(36%)	
	< 1 cm	231(31%)	29(18%)	21(32%)	26(19%)	4(10%) 310(24%)	
	suboptimal	387(47%)	11(7%)	27(41%)	17(12%)	4(10%) 110(8%)	
	Total	824	160	64	136	44	1311
BRCA1 and BRCA2 mutation status	Mutation status known but not identified (%)	1(< 1%))	0	1(3%)	0	0	2(< 1%)
	BRCA1 Carriers (%)	68(14%)	0	2(6%)	0	0	70(12%)
	double BRCA1/BRCA2 carrier (%)	4(< 1%)	0	0	0	0	4(< 1%)
	BRCA2 carriers (%)	35(7%)	0	1(3%)	0	0	36(6%)
	Non carriers (%)	370(76%)	44(100%)	31(89%)	25(100%)	6(100%)	476(80%)
	Carriers of variants of unknown significance	10(2%)	0	0	0	0	10(< 2%)
	Total BRCA mutation carrier	108	0	4	0	0	112
Survival rate	Alive	386(32%)	227(83%)	45(45%)	148(59%)	63(73%)	869(45%)
	Deceased	830	47	56	107	23	1063
	Total	1216	274	101	255	86	1932
5-years survival rate	Alive	353(34%)	138(80%)	46(54%)	118(57%)	34(65%)	689(44%)
	Deceased	678	35	39	89	18	859
	Total	1031	173	85	207	52	1548
Response to treatment^a^	sensitive	421(68%)	27(73%)	29(60%)	16(44%)	4(57%)	497(66%)
	resistant	201	10	19	20	3	253
	Total	622	37	48	36	7	750
Chemotherapy before first progression	none	22(2%)	35(14%)	8(67%)	15(6%)	37(53%)	117(7%)
	carbo+taxol	955(81%)	184(74%)	66(67%)	195(82%)	31(40%)	1431(78%)
	cispl+taxol	71(62%)	8(3%)	10(11%)	6(3%)	0	95(5%)
	platinum	63(5%)	14(6%)	7(7%)	13(5%)	3(4%)	100(6%)
	platinum+other	38(3%)	6(2%)	5(6%)	6(2.5%)	1(1%)	56(3%)
	taxol	3(< 1%)	0%	0	0	0	3(< 1%)
	other	17(1%)	(< 1%)	0	3(1%)	1(< 1%)	22(1%)
	Total	1169	248	96	238	73	1824
Long term survivors (> 10 years)	yes	73(8%)	38(45%)	10(16%)	23(18%)	7(23%)	151(13%)
	no	818	47	54	102	23	1044
	Total	891	85	64	125	30	1195

Borderline ovarian tumor cases were excluded

*N* number of case, *carbo* carboplatinum, *cispl* cisplatinum, *BOT* Borderline Ovarian Tumor, *HGSC* High-Grade Serous Carcinoma

^a^patients with platinum-based treatment

In particular, data on *BRCA1* or *BRCA2* mutations pertained to the presence of a mutation in either of these genes or both, or was recorded as non-carriers when no pathogenic mutation was found after targeted or full gene testing. Testing was done locally, often in genetic clinic and counselling clinics, although the original file were not available only the transcription from the hospital clinical files. Type of genetic testing or specific type of mutation was not requested. Cases carrying a variant of unknown significance and cases carrying double BRCA1 and BRCA2 mutations were excluded from survival analysis due the limited number of cases in these 2 categories.

Radiotherapy treatment and imaging results were coded as positive or negative. Progression was defined according to the Gynecologic Cancer InterGroup (GCIG), the earliest date between CA125 rise and objective clinical progression. The definition of CA125-associated recurrence was not the same in all institutions. When CA125 data were available, we defined the CA125 progression date as described by the GCIG: the first date when CA125 levels rose two times above the upper limit of the reference (35 U/ml) or nadir [7]. For patients with post-operative measurable RD, response to treatment was defined as first progression if it occurred within 6 months of the end of first-line treatment (debulking surgery and chemotherapy). Information on chemotherapy and radiation treatments included the type of drug and date of treatment initiation and completion. The cohort showed a relatively homogenous treatment type with 83% treated with a combination of platinum-taxane (carboplatinum or cisplatinum and docetaxel or paclitaxel). Platinum alone was used in 6% of cases and other agents in 4% of cases and included gemcitabine, cyclophosphamide, doxorubicine, topotecan or procytox (Table 2). The remaining 7% did not receive chemotherapy treatment. Radiotherapy was recorded in 3.4% of all patients, but mainly restricted to HGSC and CCC patients.

### TMA and immunohistochemistry-supported histotyping

The histotype of FFPE samples was reviewed by a central pathologist at the CRCHUM and confirmed on the constructed TMA by a second pathologist using the COSPv3 8-marker panel [8]. A process further referred to a two-step central pathology review. Cases with discordant original and predicted histotype were subjected to arbitration by biomarker-assisted review [8]. When no FFPE sample was available, the original histotype was used for the study. Tumor stage and cytoreduction were scored according to criteria from the Federation of Gynecologists and Obstetricians [9].

We also noticed that more than 10% of collected FFPE blocks of LGSC and MC did not contain representative proportions of malignant tumor cells but rather borderline tumor cells. Because only one block per case was available for review, we could not confirm the malignant diagnosis of the patient and did not include these cases in this study. The TMA with mucinous tumors was additionally stained with the Müllerian cell lineage marker PAX8 and the intestinal marker SATB2 [10]. Twenty-three cases of MC stained positively for PAX8 (11 focal and 12 diffuse) and 3.3% (*n* = 2/61) stained positive with SATB2. No case was deemed to be a metastatic intestinal carcinoma, which would have been indicated by absence of PAX8 expression in combination with presence of SATB2 expression.

### Statistical analysis

Kruskal-Wallis and Chi-square tests were used respectively to assess the association between continuous and categorical variables or two categorical variables. Disease-specific survival analyses were evaluated with Kaplan-Meier curves coupled with the log-rank test. To estimate the hazard ratio (HR) we used Cox proportional regression analyses. To facilitate comparisons with previous analyses, we used Kaplan-Meier or Cox regression analyses recognizing that cumulative incidence estimates and competing risk regression analyses may be more appropriate. Response to treatment and long-term survival were evaluated by logistic regression and odds ratio (OR). Significance level was set at *p* < 0.05. All statistical analyses were performed using SPSS, version 21.

## Results

### Histopathology review and reclassification of the COEUR cohort

Since EOC encompasses distinct histopathologies, we organized the analysis of the ovarian carcinoma cohort by histotype. As previously described by Köbel et al. [8], the most common histotype revision were cases reclassified from EC to HGSC (*n* = 43) and vice versa (*n* = 14), and from original HGSC to LGSC (*n* = 30) and vice versa (*n* = 19). A relatively significant proportion of MC cases were also reclassified to EC (*n* = 10). This histotyping exercise confirmed that CCC and HGSC were the two histotypes with the lowest reclassification rates at 5% (Table 3).

**Table 3:** Re-classification of histotypes

Original classification:	Revised Classification								Total original
	BOT	HGSC	EC	LGSC	CCC	MC	other	unknown	
HGSC	1	1131 (95%)	14	30	9	2	3	1	1191
EC	1	43	267 (84%)	0	0	2	3	1	317
LGSC	9	19	0	69 (72%)	0	0	0	0	96
CCC	1	4	4	0	248 (96%)	0	0	0	257
MC	10	6	10	0	0	84 (73%)	6	0	116
other	1	7	1	1	1	0	11(55%)	0	20
unknown	2	36	0	3	1	0	0	6 (NA)	48
Total after reclassification	25	1246	296	102	259	88	21	8	2045

*(%)* represent the % of original cases correctly classified, *BOT* Borderline Ovarian Tumor, *HGSC* High-Grade Serous Carcinoma, *EC* Endometrioid Carcinoma, *LGSC* Low-Grade Serous Carcinoma, *CCC* Clear Cell Carcinoma, *MC* Mucinous carcinoma

The reclassified histotype distribution followed the population frequency of ovarian cancer: HGSC (61%), LGSC (3%), EC (15%), MC (4%) and CCC (13%) (Table 2) [11]. A significant difference in patient age at diagnosis was observed among histotypes with HGSC patients being the oldest and MC the youngest of the non-serous cases (Kruskal-Wallis *p* < 0.001, test). The 5-year survival and the long-term survivor rates were also quite different among histopathologies (Table 2) and in accordance with the rates described in the literature [12]. The exceptions were EC and CCC, which showed higher survival rates (80% and 57%) compared to reports of 60% and 40%, respectively. This discrepancy could be explained by the correct reclassification of EC to HGSC, as previously reported [8, 13].

**Fig. 1:**
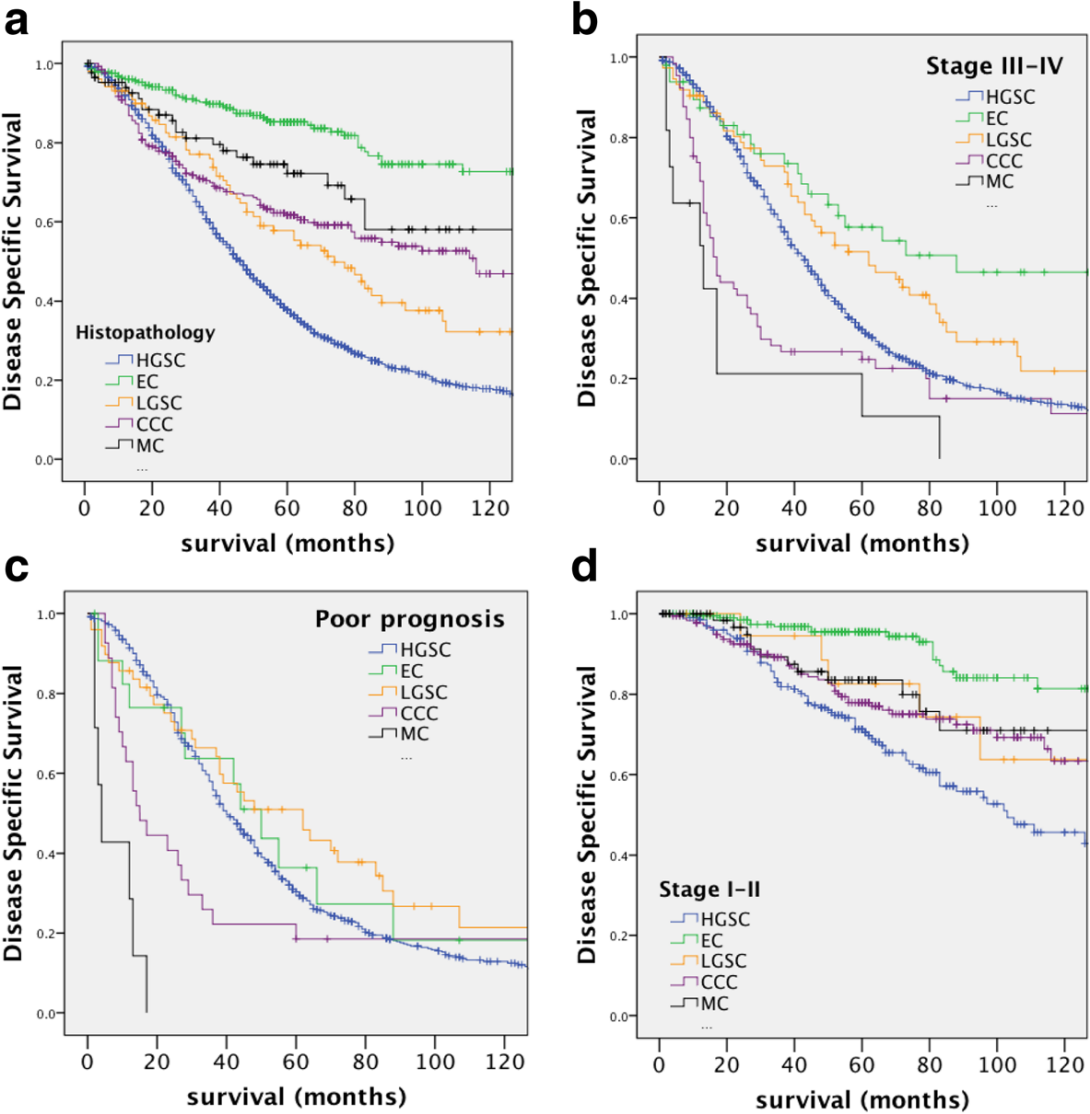
Kaplan-Meier survival curves of disease-specific survival by EOC histotypes. **a** All five histotypes (HGSC = 1246, EC = 274, LGSC = 101, CCC = 255, MC = 86). **b** Comparison of all histotypes of advanced stage (stage III and IV) (HGSC = 940, EC = 49, LGSC = 73, CCC = 65, MC = 11). **c** Comparison of all histotypes of advanced stage cases with residual disease (RD) (poor prognosis) (HGSC = 621, EC = 18, LGSC = 49, CCC = 27, MC = 7). **d** Comparison of all histotypes of low stage diseases (stage I and II). Significance (p) is indicated by log-rank (HGSC = 206, EC = 214, LGSC = 20, CCC = 178, MC = 72)

As expected based on independent reports [2, 14], HGSC cases have the lowest 5-yr and overall disease specific survival (Fig. 1a and Table 2). MC and CCC share similar survival profile (Fig. 1a) and EC cases showed the best outcome (83% survival).

### Cohort characteristics—Disease stage and cytoreduction

The proportion of non-serous histotypes with advanced stage disease (stage III-IV) was lower than other histotypes (< 25%) particularly for EC and MC histotypes as previously reported [15]. When patients were restricted to those with advanced disease (stages III and IV), the difference in overall disease-specific survival rates between HGSC and EC (30% and 53%, HR = 0.19 (0.14–0.26, 95% CI)) was drastically decreased as compared to the rate between these two histopathologies across all stages (32% and 81%, respectively; HR = 0.51 (0.34–0.77) 95% CI) Table 4 and Fig. 1b). At advanced stage, CCC and MC patients had the highest risk of death with a median survival of 17 and 13 months respectively (Table 4 and Fig. 1b). However, when patients were evaluated with presence of residual disease in advanced stage disease (further referred as “poor prognosis” disease), the difference in survival between HGSC and EC was no longer significant (21% and 33% respectively, *p* = 0.575; Table 4 and Fig. 1c). CCC and MC remained as the most deadly diseases in this category (Fig. 1c and Table 4). However, the MC cases were too few (*n* = 7) and the analysis of the MC “poor prognosis” cases should be considered with caution.

**Table 4:** Univariate Cox regression model of histotypes on disease specific survival

Histotype	All cases					Advanced stage					Poor prognosis					Low stage				
	Hazard	95.0% CI		*n*		Hazard	95.0% CI		*n*		Hazard	95.0% CI		*n*		Hazard	95.0% CI			
	Ratio	Lower	Upper		*p* value	Ratio	Lower	Upper		*p* value	Ratio	Lower	Upper		*p* value	Ratio	Lower	Upper	*n*	*p* value
HGSC				1216	reference				940	reference				621	reference				206	reference
EC	0.19	0.14	0.26	274	**0.000**	0.51	0.34	0.77	49	**0.001**	0.85	0.48	1.51	18	0.575	0.20	0.12	0.34	214	0.000
LGSC	0.62	0.47	0.81	101	**0.001**	0.67	0.50	0.91	73	**0.009**	0.72	0.50	1.03	49	0.069	0.68	0.31	1.48	20	0.332
CCC	0.51	0.41	0.62	255	**0.000**	1.63	1.24	2.16	65	**0.001**	1.54	1.00	2.36	27	**0.050**	0.62	0.43	0.89	178	**0.01**
MC	0.35	0.23	0.53	86	**0.000**	2.95	1.58	5.52	11	**0.001**	13.82	6.41	29.79	7	**0.000**	0.48	0.26	0.88	72	**0.017**

Bold values highlights the adjusted statistically significant difference with the reference category

*EOC* epithelial ovarian cancer, *n* number of patients with follow-up, *CI* confidence interval, low stage: patients with stage I and II, advanced stage: patients with stage III and IV, *HGSC* High-Grade Serous Carcinoma, *EC* Endometrioid Carcinoma, *LGSC* Low-Grade Serous Carcinoma

Among low stage diseases (stage I-II), there are no significant survival differences between HGSC and LGSC (*p* = 0.332, Table 4). HGSC and LGSC histotypes were the most deadly (62% and 65% survival, respectively, Fig. 1d) and EC remained the histopathology with the best prognostic outcome with a median survival of 15 years and a rate of 93% at 5-years (Fig. 1d).

**Fig. 2:**
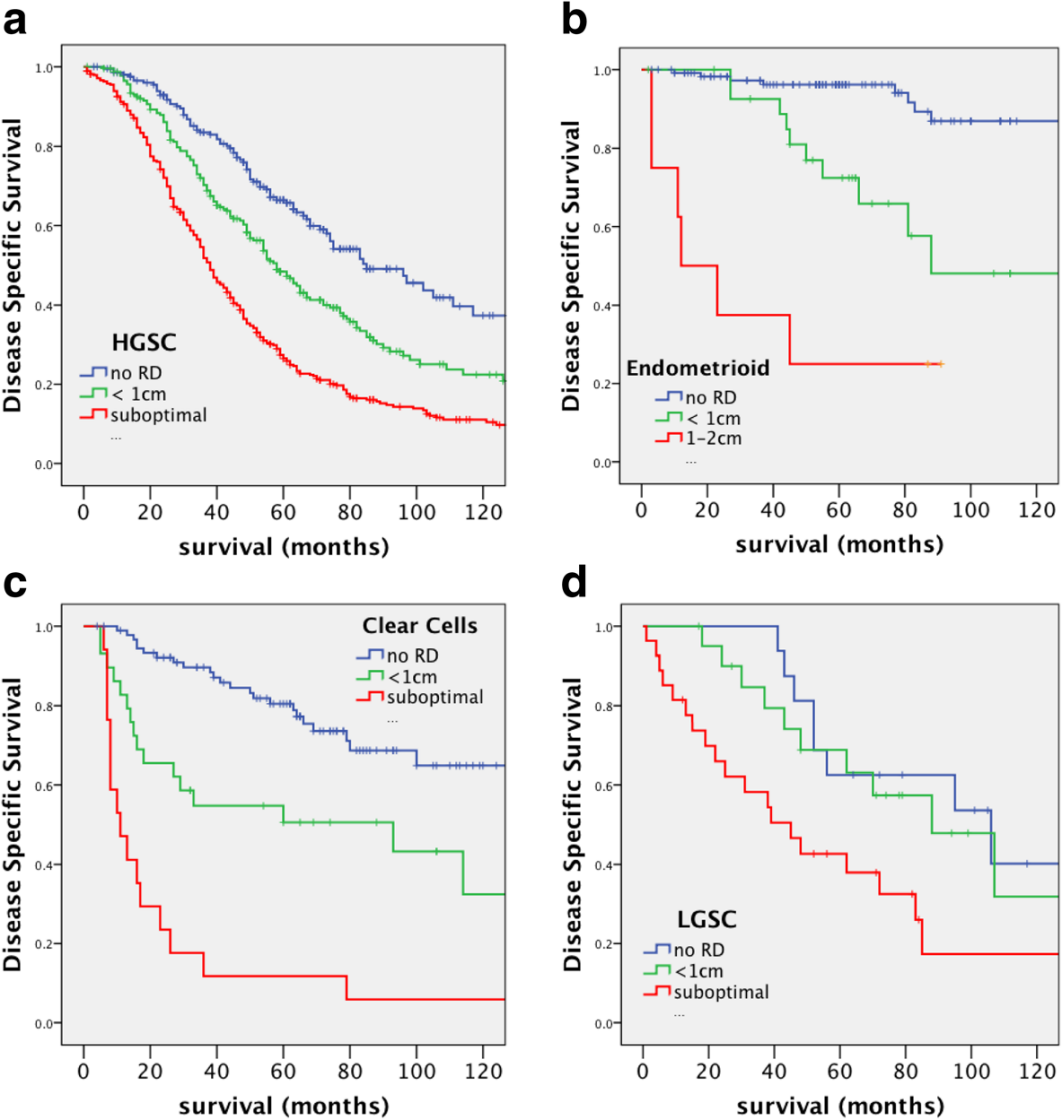
Kaplan-Meier survival curves of disease-specific survival stratified by residual disease. **a** Comparison of high-grade serous carcinoma (HGSC) with different amounts of RD (none = 206, < 1 cm = 230, suboptimal = 347). **b** Comparison of endometrioid carcinoma (EC) with different amounts of RD. (none = 119, < 1 cm = 29, suboptimal = 10) (**c**). Comparison of clear cell carcinoma (CCC) with different amounts of RD (none = 93, < 1 cm = 26, suboptimal = 17). **d** Comparison of low-grade serous carcinoma (LGSC) with different amounts of RD (none = 16, < 1 cm = 21, suboptimal = 27). Significance (p) is indicated by log-rank

The prognostic value of cytoreduction alone as a risk factor for disease specific survival was more pronounced in non-serous cases (Table 5 and Fig. 2). Residual miliary tumor was predominantly observed in serous cases (80%) and categorized as a suboptimal cytoreduction. The survival rate for EC cases with no RD was 92% compared to 25% for suboptimal cytoreduction, whereas the CCC cases with suboptimal cytoreduction showed only 7% survival rate compared to 72% with no RD. By contrast, the survival of patients in LGSC cases with no RD versus those with cytoreduction to ≤1 cm was surprisingly similar (Fig. 2d). A significant difference was only seen between LGSC patients with optimal and suboptimal cytoreduction (HR = 2.40 (1.08–5.36, 95% CI), *p* = 0.033, Table 5). The MC cases with RD were too rare for a rigorous analysis and the only 4 cases with suboptimal RD died within a year.

**Table 5:** Univariate Cox regression model of clinical parameters on disease specific survival in different EOC

Residual disease	HGSC					EC					CCC					LGSC				
	Hazard	95.0% CI		*n*		Hazard	95.0% CI		*n*		Hazard	95.0% CI		*n*		Hazard	95.0% CI		*n*	
	Ratio	Lower	Upper		*p* value	Ratio	Lower	Upper		*p* va lue	Ratio	Lower	Upper		*p* value	Ratio	Lower	Upper		*p* value
None				206	reference				119	reference				93	reference				16	reference
< 1 cm	1.72	1.31	2.23	230	**0.000**	3.80	1.63	8.84	29	**0.002**	2.24	1.17	4.29	26	**0.015**	1.10	0.45	2.72	21	0.835
Suboptimal	2.90	2.28	3.70	347	**0.000**	15.03	5.72	39.47	10	**0.000**	8.30	4.38	15.76	17	**0.000**	2.40	1.08	5.36	27	**0.033**
Stage																				
I				70	reference				140	reference				124	reference				13	reference
II	1.25	0.77	2.03	136	0.376	1.91	0.80	4.61	73	0.147	2.36	1.32	4.24	54	**0.004**	1.32	0.29	5.91	7	0.719
III	3.17	2.09	4.81	820	**0.000**	6.48	3.04	13.82	44	**0.000**	8.15	4.93	13.48	58	**0.000**	2.73	0.98	7.63	68	0.056
IV	4.61	2.93	7.26	120	**0.000**	NA	NA	NA	5	**NA**	19.35	7.57	49.43	7	**0.000**	7.11	1.87	27.00	6	**0.004**

Bold values highlights the statistically significant difference with the reference category

*NA* non applicable number of cases too low, *n* number of patients with follow-up, *HR* Hazard ratio, *EOC* epithelial ovarian cancer, *CI* confidence interval, *HGSC* High-Grade Serous Carcinoma, *EC* Endometrioid Carcinoma

### Cohort characteristics—BRCA

As 20–25% of HGSC are associated with *BRCA1*/*BRCA2* germline and somatic mutations [16, 17] we requested information from each participating site on mutation status of these genes. This variable was not available for the majority of patients, particularly in non-serous cases since mutation testing is mostly undertaken when a hereditary factor was suspected. In addition, *BRCA* mutation testing is sometimes targeted to only a few mutation sites found to recur in specific populations, and thus not always fully sequenced or assessed for longer deletions. For this reason, cases identified as a *BRCA* non-carrier genotype in our cohort were likely over-estimated. Nonetheless, we collected 112 cases of known mutation *BRCA* mutation carriers and 476 nominally non-carrier cases (Table 2). The proportion of *BRCA* mutation carriers was 19% of all tested cases, and 22% of HGSC. Indeed, 96% of *BRCA* mutations (*n* = 108) were detected in HGSC, while four cases were classified as LGSC.

**Fig. 3:**
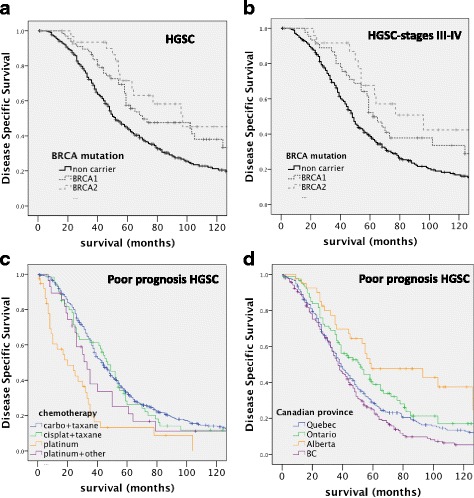
Kaplan-Meier survival curves of disease-specific survival stratified by *BRCA* status (**a-d**). **a** Comparison of HGSC patients by *BRCA* mutation. Mutation non-carriers (black, *n* = 351), *BRCA1* mutation positive (dark grey, *n* = 67) and *BRCA2* mutation positive (dashed light grey, *n* = 34). **b** Comparison of HGSC patients by *BRCA* mutation in advanced stage disease. Mutation non-carriers (black, *n* = 293), *BRCA1* mutation positive (dark grey, *n* = 45) and *BRCA2* mutation positive (dashed light grey, *n* = 26). **c** Kaplan-Meier survival curves of disease-specific survival stratified by chemotreatment type. Comparison of poor prognosis HGSC patients by chemo-treatment: carboplatinum/taxane (blue, *n* = 469); cisplatinum/taxane (green, *n* = 60); platinum alone (gold, *n* = 38); platinum + another agent (violet, *n* = 28). **d** Kaplan-Meier survival curves of disease-specific survival stratified by source site. Comparison of provincial origins of poor prognosis HGSC patients in the COEUR cohort (Quebec = 275, Ontario = 89, Alberta *n* = 41, British Columbia = 210). Significance (p) is indicated by log-rank

As expected, the age of HGSC patients with *BRCA* mutation was, on average, lower than the age of non-carrier HGSC patients (53 and 60 years, respectively, *p* < 0001, Mann-Whitney U test). Patients with a *BRCA2* mutation also presented more often with advanced disease compared to patients with a *BRCA1* mutation (69% compared to 80%, *p* = 0.012, Chi-Square) as previously observed by others in larger cohorts [18, 19]. In HGSC cases treated with a platinum-based regimen, we observed a significant association between the presence of any *BRCA* mutation and survival, with a median survival of 96 months in the mutated group as compared to 61 months in the non-carrier group (HR = 0.56(0.42–0.76 95%CI), *p* < 0.001, Table 6; Fig. 3a). When stratified to advanced stage disease, the *BRCA* mutation carrier status remained a significant prognostic variable (HR = 0.57 (%95 CI 0.41–0.80), *p* = 0.001, Table 6 and Fig. 3b) but not when stratified to low stage disease (*p* = 0.82, Table 6). The improved outcome of mutation carrier women was slightly higher in patients with mutated *BRCA2* than with mutated *BRCA1* (Fig. 3a and Table 6). This trend was even stronger in “poor prognosis” cases, where only *BRCA2* carriers remained of significant survival advantage (HR = 0.39 (%95 CI 0.20–0.77), *p* = 0.006) with a median survival of 77 months compared to 58 months for *BRCA1* carriers and 45 months in the non-carrier group.

**Table 6:** Univariate Cox regression model of clinical parameters on disease specific survival in stratified high-grade serous carcinoma (HGSC)

	All HGSC					Advanced stage HGSC					Poor prognosis HGSC					Low Stage HGSC				
	Hazard	95.0% CI				Hazard	95.0% CI				Hazard	95.0% CI				Hazard	95.0% CI			
	Ratio	Lower	Upper	n	p value	Ratio	Lower	Upper	n	p value	Ratio	Lower	Upper	n	p value	Ratio	Lower	Upper	n	p value
*BRCA* mutation carrier status^a^																				
Non carrier				351	reference				293	reference				236	reference				61	reference
BRCA1 carrier	0.62	0.44	0.88	67	**0.007**	0.65	0.44	0.95	45	**0.029**	0.79	0.51	1.20	32	0.268	0.92	0.40	2.11	21	0.849
BRCA2 carrier	0.42	0.24	0.72	34	**0.002**	0.41	0.22	0.76	26	**0.004**	0.39	0.20	0.77	21	**0.006**	0.80	0.19	3.44	7	0.766
All mutated vs no mutation	0.56	0.42	0.76	472	**0.000**	0.57	0.41	0.80	375	**0.001**	0.64	0.44	0.93	294	**0.02**	0.82	0.37	1.81	85	0.78
Chemotherapy treatment type																				
Carboplatinum+taxane				940	reference				727	reference				469	reference				167	reference
Cisplatinum+taxane	1.14	0.87	1.50	71	0.348	0.63	0.44	0.89	67	0.728	1.11	0.83	1.49	60	0.495	NA	NA	NA	4	NA
Platinum	1.86	1.37	2.52	63	**0.000**	0.42	0.24	0.73	46	**0.000**	2.65	1.83	3.83	38	**0.000**	1.65	0.71	3.84	13	0.245
Platinum+other	1.93	1.35	2.75	38	**0.000**	0.56	0.42	0.76	33	**0.005**	1.44	0.94	2.19	28	0.093	NA	NA	NA	3	NA
Provinces																				
Quebec				584	reference				472	reference				275	reference		Lower	Upper	NA	
Ontario	0.80	0.66	0.98	195	**0.034**	0.69	0.54	0.88	119	**0.003**	0.77	0.58	1.03	89	0.074	NA	NA	NA	NA	NA
Alberta	0.71	0.56	0.91	120	**0.007**	0.73	0.56	0.96	88	**0.025**	0.51	0.34	0.78	41	**0.002**	NA	NA	NA	NA	NA
Manitoba	0.46	0.15	1.45	10	0.185	0.20	0.03	1.45	7	0.111	NA	NA	NA	6	NA	NA	NA	NA	NA	NA
British Columbia	0.99	0.84	1.17	307	0.917	1.02	0.86	1.21	248	0.841	1.21	0.99	1.47	210	0.064	NA	NA	NA	NA	NA

Bold values highlights the statistically significant difference with the reference category

*NA* non applicable, number of cases too low, *n* number of patients with follow-up, *HR* Hazard ratio

^a^ platinum-based patients only, low stage: disease stage I and II, advanced stage: disease stage III and IV

Interestingly, we also observed a better long-term survival rate (> 10 years) among *BRCA1* or − *2* mutation carriers compared to the non-carrier group (21% vs 11%, OR = 0.432, 95%CI (0.21–0.90), *p* = 0.024, *n* = 319). In advanced stages patients, *BRCA1* or *BRCA2* mutation was still associated with long-term survival (22% vs 10%, OR = 0.41, 95% CI (0.18–5.78), *p* = 0.94, *n* = 283) but no more among poor prognosis patients (*p* = 0.27).

### Cohort characteristics—Chemotherapeutic treatments

We compared the disease-specific survival rates among the most common types of chemotherapeutic regimens. Only a few patients were treated with a regimen other than platinum as their first adjuvant chemotherapy (Table 2) and, therefore, were excluded from this analysis. Treatment regimens that were analyzed included carboplatinum/taxane, cisplatinium/taxane, platinum alone or platinum + another non-taxane agent. HGSC patients subjected to both platinum plus taxane treatments had better survival compared to patients treated with platinum alone or platinum + another agent (*p* < 0.001 and Table 6). In “poor-prognosis” HGSC cases, platinum + another agent regimen was no more significantly less effective than a platinum/taxane regimen (*p* = 0.093, Table 6 and Fig. 3c).

Treatment resistance in patients with RD was defined as progression within 6 months of the end of first treatment*,* that is following surgery and treated with a chemotherapy agent as per the standard clinical definition [20]. We restricted the analysis to platinum-based treated patients because of the limited number of non-platinum treated cases. There was no difference in treatment response rate between LGSC and EC (Table 2). Only CCC showed a significantly lower response to treatment compared to type other histotypes (OR = 2.73 (95%CI 1.42–5.26), *p* = 0.003). Treatment resistance could not be accurately analyzed in the MC histotype (*n* = 7). The small number of non-serous cases with RD limited this analysis, and interpretation of our findings will require a larger cohort.

Among the known prognostic factors, *BRCA1* or *BRCA2* mutation carrier status was a favorable factor relative the non-carrier status for response to treatment in HGSC cases treated with surgery and platinum-based chemotherapy. We observed that 35% of cases had progressed within 6 months in the non-carrier group (*n* = 248) compared to 21% in the *BRCA* mutation carrier group (*n* = 59) (OR = 0.44, 95% CI 0.22–0.89, *p* = 0.022, Table 7). A similar result was obtained in evaluating advanced stage patients although less significant (36% vs 16%, OR = 0.49, (95%CI 0.24–1.00), *p* = 0.042, Table 7). When BRCA mutation was stratified in BRCA1 or BRCA2 mutation status, BRCA2 alone was no more associated with better response to treatment (Table 7). However, due to the small number of *BRCA2* carriers with RD, this result should be confirmed in a larger cohort.

**Table 7:** Univariate logistic regression model of clinical parameters on treatment response in stratified high---grade serous carcinoma (HGSC)^a^

	All HGSC					Advanced stage HGSC				
	Hazard	95.0% CI				Hazard	95.0% CI			
	Ratio	Lower	Upper	*n*	*p* value	Ratio	Lower	Upper	*n*	*p* value
*BRCA* mutation carrier status										
Non carrier				247	reference				228	reference
BRCA1 carrier	0.29	0.11	0.77	38	**0.013**	0.34	0.13	0.91	32	**0.031**
BRCA2 carrier	0.72	0.27	1.89	22	0.499	0.73	0.27	1.94	21	0.524
All mutated vs no mutation	0.44	0.22	0.89	307	**0.022**	0.49	0.24	1.00	281	**0.042**
Provinces										
Quebec				256	reference				239	reference
Ontario	0.47	0.27	0.82	93	**0.008**	0.40	0.21	0.74	80	**0.00**
Alberta	0.22	0.08	0.58	46	**0.002**	0.19	0.07	0.55	40	**0.002**
Manitoba	NA	NA	NA	8	0.999	NA	NA	NA	5	NA
British Columbia	0.95	0.63	1.44	160	0.808	0.92	0.60	1.40	155	0.689

Bold values highlights the statistically significant difference with the reference category

^a^ platinum-­-based patients only

### Participating biobanks of the COEUR cohort

Twelve Canadian biobanks representing the five most populated provinces contributed samples to the COEUR repository (Table 8). Due to sample availability in each biobank, the five Canadian provinces are not equally represented in the COEUR cohort. The cohort may not necessarily represent the Canadian demography of each province. For example, Quebec cases represent almost half of the entire cohort, whereas cases from the most populous Canadian province, Ontario, represent only 16% of the cohort. Also, the relatively new establishment of the Manitoba Ovarian Biobank Program (MOBP) in 2011 resulted in fewer cases from this province.

**Table 8:** Distribution and clinical characteristcs of the COEUR cases per Canadian province

	Location of tumor bank	Quebec	Ontario	Manitoba	Alberta	British Columbia	Total
	Total cases	976	330	242	17	479	2046
Histotype	BOT	9	4	7	0	3	23
	HGSC	603	199	120	10	314	1246
	EC	132	56	31	3	73	295
	LGSC	52	24	19	0	8	103
	CCC	123	37	36	1	62	259
	MC	43	1	28	2	14	88
	other	14	8	1	1	4	29
Age (mean years)^a^	at diagnosis	63	60	61	61	62	62
	at end of follow-up	66	64	65	66	66	65
Year of diagnosis (range)		1992–2015	1996–2013	2012–2015	1998–2015	1998–2013	1992–2015
Follow-up (mean, months)		54(0–270)	51(0–168)	32(9–49)	49(0–181)	47(0–173)	48(0–270)
Survival rate^a^	Disease specific survival	30%	41%	73%	36%	29%	33%
	5-years survival rate	31%	40%	NA	42%	33%	34%
	Long Term Survival rate [> 10 years]	9%	7%	NA	12%	6%	8%
Treatment type	none	47	5	55	0	23	130
	carboplatin+taxol	690	238	160	15	351	1454
	cisplatin+taxol	40	28	9	0	20	97
	platinum	21	21	15	0	45	102
	platinum+other	47	2	1	0	6	56
	taxol	3	0	0	0	0	3
	other	15	6	0	0	1	22
	Total	863	300	240	15	446	1864
	% response to treatment^a^	69%	84%	90%	91%	71%	75%
BRCA mutation carrier status	non carriers	306	27	6	9	137	485
	carriers	58	16	2	4	33	113
	total	370	33	8	13	170	594
	% carriers	16%	48%	25%	31%	19%	19%

*NA* non applicable, *BOT* borderline tumor, *HGSC* High-Grade Serous Carcinoma, *EC* Endometrioid Carcinoma, *LGSC* Low-Grade Serous Carcinoma, *CCC* Clear Cell Carcinoma, *MC* Mucinous Carcinoma

^a^ HGSC patients only

The distribution of death risk factors (age, histotype, response to treatment, *BRCA* mutation status) among the different contributory banks for each case by province was heterogeneous (Table 8). In Quebec, we also noticed more cases treated with platinum and another agent as an alternative to platinum + taxane, while other provinces provided more cases treated with platinum as a single agent.

To date, the literature has no reports on the ovarian cancer survival rate among Canadian provinces compared with the province of Quebec due to a lack of a Quebec-based registry. Moreover, current statistics often do not consider patient group according to metric such as stage, cytoreduction, and histopathology, when surveying survival of ovarian cancer patients. To compare EOC death rates among provinces, we limited our analysis to cases with either advanced stage HGSC or the “poor prognosis” patients. Manitoba was not included due to the small number of cases (n = 7 for advanced HGSC patients). The 5-year and overall survival was better in Ontario and Alberta compared to Quebec (OR = 0.56 (95% CI 0.36–0.87) *p* = 0.010, and Table 8), although less significant when Ontario were restricted to “poor prognosis” patients (*p* = 0.074, Table 6 and Fig. 3d).

## Discussion

The COEUR has enabled the assembly of many retrospective cases (> 2000) in a relatively short period of time (7 years). This network has ensured the standardization of the clinical data after collection and a long-term follow-up. Moreover, to facilitate this endeavor, a single material transfer agreement was established and signed by all institutions, which provided a consistent legal and ethics policy for subsequent users to access the centralized collection of specimens and clinical data. To date 25 national and international studies got access to the cohort [5, 8, 21–23]. The cohort contains more than 100 LGSC and 80 MC cases, the rarest of all EOC histotypes. Although these numbers are small, most biomarker studies analyzing these histotypes have rarely described a cohort of this size containing the least common histotypes, particularly after contemporary histopathology review. Indeed, of significant value is the two-step central pathology review of all FFPE cases with application of current diagnostic immunohistochemical markers adding an objective molecular layer. This rigorous review of all cases by the same pathologists standardized and corrected the inaccuracies of previous or out-dated pathology records and resulted in reclassifying a significant number of EC cases to HGSC, supported by improved outcome stratification. A significant proportion of MC cases were also reclassified to EC or metastatic disease, which in turn highlighted the rarity of advanced stage MC cases. The possibility of contamination of the cohort by cases with metastatic intestinal adenocarcinomas from the colon and appendix amongst presumed MC cases, was minimized by immunohistochemistry phenotyping. This qualitative revision of cases ensures a high standard for a shared research resource.

The COEUR cohort has some limitations. One drawback is the collection of cases based on specimen availability several years after initial collection, which may not represent the overall biobank population, nor even the provincial ovarian cancer population. Another limitation of the COEUR cohort, even if it represents the demographic situation of uncommon histotypes, is the under-representation of rare histotypes at advanced stages. This will limit the ability to reach sufficient statistical power in biomarker studies. For example, we were not able to analyze the cytoreduction impact on survival in MC cases or the treatment resistance in patients with RD. An alternative to this limitation could be the specific collection of rare cases. Another important point is that only one sample per patient was collected, indicating that review of the complete case was not possible for the majority of cases. Another disadvantage of a multicenter repository is the different standards applied for subjective clinico-pathological parameters, such as disease stage, cytoreduction amount, and treatment regimen. For example, some institutions did not include rising CA125 levels as an indicator of disease progression, and perhaps allowed a less accurate progression time and less regular follow-up than other institutions.

In this report, we have described the clinical characteristics of the COEUR cohort, which show similarities to the characteristics of the general ovarian cancer population as reported in the literature. The proportion of non-serous histotypes with advanced stage disease was lower than other histotypes (< 25%), particularly with MC cases, as previously reported [15, 24]. The 5-year survival and the long-term survival rates of each histotype are in accordance with the rates described in the literature, particularly when histotypes were diagnosed with the current histological criteria [2, 8, 13, 14].

Different clinical outcomes were observed among histotypes; when not adjusted to prognostic clinicoparameters such as stage and cytoreduction, HGSC cases demonstrate the worst outcome compared to the other histotypes. This data is particularly noteworthy as individual biomarker studies, that have been limited in statistical power due to low incidence of non-serous EOC, are prone to consider risk factors and prognostic biomarkers for all ovarian tumors as one group. Since many biomarkers have been associated with specific histotypes, studies evaluating outcome in cohorts comprising all histotypes are likely confounded by inherent differences in the outcomes of specific histotype rather than the biomarker itself [2]. Furthermore, and as seen here, stratification of histotypes by stage and amount of RD should also be considered in biomarker prognostic studies (Fig. 1b-h). Indeed, when adjusted for advanced stage or suboptimal cytoreduction, MC and CCC rather than HGSC appears to be the deadliest disease with 25% survival consistent with independent reports [13, 25–28]. Although EC is reported to have a similar survival outcome in advanced disease as compared to HGSC [11], an analysis of the COEUR cohort revealed a strong survival advantage for EC and this observation could be attributed to the reclassification of some EC to HGSC cases.

The COEUR cohort has a good representation of the five major histotypes without major bias, and hence, is a useful resource for biomarker validation. Indeed, some studies have already used these specimens to validate a diagnostic biomarker algorithm and have used samples as an inter-laboratory quality control for such markers, which are now routinely used in Canadian and British pathology laboratories [8, 29]. In one study, COEUR samples were used to optimize p53 immunohistochemistry and achieved a 97% accuracy in predicting *TP53* mutation status in ovarian carcinomas, which is now widely adopted in pathology departments [21]. The COEUR cohort has also validated markers of prognostic value that are currently under further investigation for their clinical utility [22].

Due to its role in the repair of double strand DNA breaks by homologous recombination, patients harboring a *BRCA* mutation and consequently a defect in homologous recombination in their tumors are more sensitive to DNA crosslinking caused by DNA-damaging-platinum-based chemotherapeutic treatments. In the COEUR cohort, all of the *BRCA* mutations were exclusively reported in serous cases. After histotype reclassification and data monitoring, we did not observe any *BRCA* mutations in non-serous cases, although these diseases are rarely tested for familial risk by DNA sequencing, HGSC have been shown to be enriched for *BRCA1/2* carriers [30]. To confirm the paucity of *BRCA* mutations in other non-HGSC histotypes in the COEUR cohort, further genetics tests are necessary for the remaining cases not sequenced.

Borderline tumors and LGSC tumors are usually not associated with *BRCA* familial risk except in rare cases [31–33]. These cases have often been identified in patients of young age (≤50 yrs) within the Jewish population and show aggressive characteristics [32, 33]. In the COEUR cohort, among the four LGSC cases with *BRCA* mutation, three cases underwent the two-step pathology review and the LGSC diagnosis was confirmed. The last case was originally diagnosed as HGSC but had only one FFPE specimen sent to the COEUR. Therefore, we were not able to review and confirm the LGSC diagnosis of this patient. Since here we collected only data for the *BRCA* germline mutation carrier status but not details about the mutation type, we are uncertain whether the same mutations are present in the tumor tissue or whether an unrelated histotype evolved in these *BRCA* mutation carriers. Lastly, we do not know the specific significance of these *BRCA* mutations and whether they unambiguously represent a pathognomonic mutation. Our data suggest that *BRCA* mutations are largely restricted to HGSC consistent with recent independent reports [30, 34].

The presence of a germline and/or somatic *BRCA* mutation has been recognized as a good prognostic factor [16, 18, 19, 35–37] in small cohorts and confirmed by Bolton et al. in a pooled analysis of 909 *BRCA1* and 304 *BRCA2* patients [18]. Our data are consistent with these reports (Fig. 2). Yang et al. have also reported a better survival for 29 *BRCA2* cases as compared to 37 *BRCA1* cases found in the TCGA database and attributed this to a longer treatment-free interval after chemotherapy treatment [38]. We also observed a better overall and long-term survival of advanced stage *BRCA* mutation carriers, particularly *BRCA2*, although this favourable outcome was not associated with a better treatment response within 6 months when *BRCA1* and *BRCA2* patients were compared. This is in contrast to a study by McLaughlin et al. [39] that reported no differences in long-term survival in *BRCA* mutation carriers. Notably, this independent report included all ovarian carcinomas histotypes, and the 5-year survival rate of the non-carrier group was greater than 50% [39]. This may have confounded non-HGSC outcomes, particularly LGSC or EC, which are largely unrelated to *BRCA* mutations. The present study is the first time where *BRCA* mutation was estimated in stratified groups of homogeneous prognostic factor in HGSC patients after double central pathological revision, confirming previous data showing a 5-yrs survival advantage of *BRCA2* patients [18, 40]. Our data show the improved long-term outcome of HGSC patients bearing germ-line BRCA mutation treated with platinum-based chemotherapy relative to HGSC non-carriers, although this advantage restricted to advanced stage disease patients. These data have potential implications and underline the need for BRCA testing for the clinical management and personalized treatment of advanced stage HGSC patients.

Finally, we observed a better disease-specific survival rate in patients from Ontario and Alberta, compared to other provinces, similarly to the pattern observed from cancer-registries in 4 Canadian provinces [41]. In fact, a study in Canadian showed that geographic location of patients could impact outcomes of ovarian cancer du differences in patient management and treatment [42]. Altogether, these observations also highlight that biomarker studies using the COEUR cohort should include verification if the value of biomarker is not biased towards case source. This is particularly important in TMA studies since pathology departments may apply different standards, and tissue preservation over time may affect biomarker stability.

## Conclusions

Our study of a well-defined ovarian cancer cohort shows the tremendous importance of studying EOC by histologic histotype and the strength of a two-step central pathological review, allowing for observation of significant differences of prognostic factors and biomarkers in a relatively small sample size as seen with *BRCA* patients. In addition, the heterogeneity of risk factors in each histotype, such as disease stage, cytoreduction, *BRCA* mutation carrier status and sample source, highlights the importance of stratifying or adjusting for these factors when evaluating the prognostic value of biomarkers. COEUR provides a validation platform for biomarker studies on a variety of biological materials with contemporary histotype information and high quality clinical data for the national and international ovarian cancer researcher community.

## Abbreviations

CCC: Clear cell carcinoma
COEUR: Canadian ovarian experimental unified resource
CTRnet: Canadian tissue repository network
EC: Endometrioid carcinoma
EOC: Epithelial ovarian cancer
FFPE: Formalin-fixed paraffin-embedded
GOC: Society of gynecologic oncology of Canada
HGSC: High-grade serous carcinoma
HR: Hazard ratio
LGSC: Low-grade serous carcinoma
LTS: Long-term survival
MC: Mucinous carcinoma
OR: Odds ratio
RD: Residual disease
TMA: Tissue microarray
